# Effects of Medicaid expansion on access, treatment and outcomes for patients with acute myocardial infarction

**DOI:** 10.1371/journal.pone.0232097

**Published:** 2020-04-23

**Authors:** Erica M. Valdovinos, Matthew J. Niedzwiecki, Joanna Guo, Renee Y. Hsia

**Affiliations:** 1 Department of Emergency Medicine, Adventist Health Ukiah Valley, Ukiah, California, United States of America; 2 Mathematica Policy Research, Oakland, California, United States of America; 3 Department of Emergency Medicine, University of California, San Francisco, California, United States of America; 4 Philip R. Lee Institute for Health Policy Studies, University of California, San Francisco, California, United States of America; Ball State University, UNITED STATES

## Abstract

**Introduction:**

Uninsured patients have decreased access to care, lower rates of percutaneous coronary intervention (PCI), and worse outcomes after acute myocardial infarction (AMI). The aim of this study was to determine whether expanding insurance coverage through the Affordable Care Act’s expansion of Medicaid eligibility affected access to PCI hospitals, rates of PCI, 30-day readmissions, and in-hospital mortality after AMI.

**Methods:**

Quasi-experimental, difference-in-differences analysis of Medicaid and uninsured patients with acute myocardial infarction in California, which expanded Medicaid through the Affordable Care Act, and Florida, which did not, from 2010–2015. This study accounts for the early expansion of Medicaid in certain California counties that began as early as July 2011. Main outcomes included rates of admission to PCI hospitals, rates of transfer for patients who initially presented to non-PCI hospitals, rates of PCI, rates of early PCI defined as within 48 hours of hospital admission, in-hospital mortality, and 30-day readmission.

**Results:**

55,991 hospital admissions between 2010–2015 met inclusion criteria. Of these, 32,540 were in California, which expanded Medicaid, and 23,451 were in Florida, which did not. 30-day readmission rates after AMI decreased by an absolute difference of 1.22 percentage points after the Medicaid expansion (95% CI -2.14 to -0.30, P < 0.01). This represented a relative decrease in readmission rates of 9.5% after AMI. No relationship between the Medicaid expansion and admission to PCI hospitals, transfer to PCI hospitals, rates of PCI, rates of early PCI, or in-hospital mortality were observed.

**Conclusions:**

Hospital readmissions decreased by 9.5% after the Affordable Care Act expanded Medicaid eligibility, although there was no association found between Medicaid expansion and access to PCI hospitals or treatment with PCI. Better understanding the ways that Medicaid expansion might affect care for vulnerable populations with AMI is important for policymakers considering whether to expand Medicaid eligibility in their state.

## Introduction

Patients without insurance experience lower quality of care and poorer outcomes after acute myocardial infarction (AMI), known colloquially as a heart attack.[1–6] Compared with privately insured patients with AMI, uninsured patients with AMI are less likely to be admitted to hospitals that perform invasive reperfusion treatments and less likely to be transferred to receive coronary revascularization.[1, 2] Patients without insurance with AMI are also less likely to receive invasive treatments such as percutaneous coronary intervention (PCI),[3] which may contribute to their higher mortality rates compared with privately insured patients after AMI.[4, 5] Invasive treatments such as percutaneous coronary intervention (PCI), also known as coronary revascularization, open up the blood vessels that are blocked in a heart attack and can be lifesaving. The mechanisms by which insurance status affects care and outcomes after AMI are not all completely understood, but might include delays in accessing care,[7] the capabilities of hospitals that uninsured patients access,[8] the likelihood of an uninsured patient being offered an expensive, invasive procedure by their care teams,[9] the likelihood of being transferred to an outside hospital,[1, 2] and access to medical care after discharge from the hospital.[10–12]

The Affordable Care Act (ACA) expanded Medicaid eligibility and dramatically changed the context for insurance coverage of vulnerable populations in 2014, with individuals earning up to 138% of the federal poverty level newly eligible for Medicaid. More than two million people, however, remain uninsured in states that did not expand Medicaid eligibility after a Supreme Court ruling that allowed states to opt out.[13, 14] At this time, 14 states have not adopted the Medicaid expansion. Early studies on the ACA’s Medicaid expansion indicate that it has led to increased access to and quality of primary care,[15, 16] improved quality of care for common surgical conditions,[17] better outcomes after cardiac surgery, [18] and a decrease in cardiovascular mortality.[19] The literature has not yet described whether Medicaid expansion has affected acute care for AMI including access to care and outcomes. A nationwide study of patients hospitalized with AMI found that patients in expansion states were more likely to have insurance after the Medicaid expansion but did not identify an association between the Medicaid expansion and markers of quality of care for AMI including revascularization therapy, or in-hospital mortality.[20] This study, however, did not include patients who were transferred or address whether access to PCI hospitals increased for patients with AMI after Medicaid expansion.

Therefore, we sought to understand whether or not expanding Medicaid eligibility through the ACA was associated with access to care, receiving PCI, and outcomes for the common, emergent condition of AMI in Medicaid-insured and uninsured patients from 2010 to 2015. We compared California, a state that expanded Medicaid, with Florida, a state that did not, to estimate the impact of the Medicaid expansion on access to PCI hospitals, rates of PCI, hospital readmissions, and in-hospital mortality for patients with AMI. PCI is an invasive, expensive, and emergent procedure, which has been shown to improve outcomes in patients with AMI if treated early.[21, 22] This study takes an important step toward understanding how the ACA impacted access to care, treatment and outcomes of vulnerable populations with AMI.

## Materials and methods

### Data

We chose to examine California, which expanded Medicaid through the ACA, as the “intervention” state and Florida, which did not expand Medicaid, as the “control” state. We chose California in part for its importance as a Medicaid expansion state; Medicaid covers one quarter of the state’s population, with more than 3.6 million adults in the state enrolling in Medicaid through the expansion.[23] In contrast Florida, which did not expand Medicaid, is home to the second highest number of adults (after Texas) who would stand to gain insurance if the state expanded Medicaid.[24] Demographic differences and practice variation exist between the states, however our study design uses a difference-in-differences approach to control for such differences, as described below.

We obtained California non-public patient discharge, hospital utilization, and financial data from the California Office of Statewide Health Planning and Development (OSHPD), which has been previously used to estimate disparities in care after AMI.[25] The OSHPD data is supplied by the state of California to the Healthcare Cost and Utilization Project (HCUP). We obtained Florida patient data from the HCUP State Inpatient Database (SID) and State Emergency Department Database (SEDD). The SID captures 97 percent of US community hospital discharges and has been previously used to study the effect of state-level insurance policy interventions on outcomes and disparities.[26] The SEDD includes emergency department (ED) visits that do not lead to admission. Both OSHPD and HCUP data include unique patient identifiers, which allowed us to track the same patients over time, across different hospitals within each state. Though we obtained the California data from OSHPD and the Florida data from HCUP, because the OSHPD data is supplied to HCUP, the data sets are equivalent. Unfortunately, there is not SID data available for Texas through HCUP for the years studied.

### Patient population

We identified patients with a primary diagnosis of AMI on visits that originated in the ED (International Classification of Diseases, Ninth Revision, Clinical Modification [ICD-9-CM] 410.x0 and 410.x1)[27] from January 1, 2010 through September 30, 2015. We chose to use a cut-off of September 30, 2015, since diagnosis classifications changed on October 1, 2015, to ICD-10-CM, and performing longitudinal analyses across these time periods has not been validated with significant coding inconsistencies.[28, 29] We included uninsured and Medicaid-insured patients, the population most affected by the expansion of Medicaid.[26, 30] We included patients ages 18–64, excluding patients aged 65 or older as these patients would likely have been covered by Medicare and minimally, if at all, affected by the expansion of Medicaid. To ensure we examined only patients with primary presentations for AMI, we only included the visit in which the patient first presents with AMI in the data set. We excluded patients who were admitted with an AMI diagnosis during the previous 12 months.[27]

For our sensitivity analysis using ST-segment elevation myocardial infarction (STEMI) patients, we defined patients as having STEMI if they had a primary discharge ICD-9 code of 410.x0, 410.x1, excluding 410.7x and 410.9x.

### Variables

While most states participating in the Medicaid expansion implemented the policy at the start of 2014, California counties began expanding Medicaid eligibility at various times, beginning as early as 2011.[31] To account for these early expansion counties, we created eight county cohorts based on the month and year of the Medicaid expansion, with discharges categorized as “post-reform” if they occurred during or after the month that county expanded Medicaid (S1 Table). Other control variables included sex, age, race, year of discharge, urbanity of patient’s home county, and comorbidities. To identify comorbidities, we used the Elixhauser comorbidity index and secondary diagnoses.[32]

### Study outcomes

We selected outcomes previously shown to be influenced by insurance coverage: likelihood of admission to a hospital with PCI capacity (“PCI hospital”),[1] likelihood of transfer for patients who initially presented with AMI to hospitals without PCI capacity,[1, 2] likelihood of undergoing PCI during index hospitalization,[9] early PCI defined as having received PCI within 48 hours of admission,[33] in-hospital mortality, and readmission within 30 days of discharge.[6] We defined receipt of PCI by procedure codes in the discharge record (ICD-9 codes 0.66, 17.55, 36.00, 36.04, 36.06, 36.07, 36.09),[25, 34–37] Following previous literature, we defined hospitals as PCI hospitals if they performed at least 4 PCIs per year.[38–40] If a patient was transferred and received PCI at the destination hospital, we considered the procedure to be performed on index hospitalization. We included transfers both from the ED and from inpatient stays. We used unique patient identifiers in the data set to track patients between hospitals, and identified patients who were transferred from the ED by the following criteria: the disposition from the ED was “transfer” and the patient had an inpatient admission observed at a separate hospital within 1 day of the ED visit, or inpatient visits with an admission listed as originating from the ED and a transfer. To identify patients transferred from an inpatient stay to another hospital we used the following criteria: inpatient visits in which the disposition was “transfer” with a second hospital admission within 1 day, or inpatient visits with an admission originating from “transfer” and the patient had a previous inpatient admission at a different hospital within 1 day.

### Study design

To estimate the effect of the Medicaid expansion on treatment and outcomes after AMI, we used a quasi-experimental, difference-in-differences design to compare likelihood of admission to a PCI hospital, likelihood of transfer for those initially presenting to hospitals without revascularization capacity, PCI rates, early PCI rates, 30-day readmissions, and in-hospital mortality after AMI in California and Florida, before and after the expansion took place. We used linear regression models to estimate the difference-in-differences. We included location (county level) and time (month) fixed effects as well as indicators for when the Medicaid expansion was in effect for a particular county. We adjusted for sex, age, race, year of discharge, county, urbanity of patient’s home county, and comorbidities, and clustered standard errors at the hospital level. The intervention, Medicaid expansion, occurred at different times depending on when the county expanded Medicaid, because of the varying dates of Medicaid expansion in California. We used eight county cohorts, which we created based on the month and year of the Medicaid expansion, with discharges categorized as “post-reform” if they occurred during or after the month that county expanded Medicaid (S1 Table). Using two-way fixed effects (location and time), along with an indicator for the treatment period, to implement a difference-in-differences regression analysis of a program with staggered start dates is a standard approach widely used in the social sciences literature.[41, 42] The overall effect of the Medicaid expansion is measured as the average effect across the multiple points in time when California counties expanded Medicaid eligibility.

The difference-in-differences approach has been used in other studies examining the effect of the Medicaid expansion.[17, 43] This design estimates the effect of a policy change by comparing the change in an outcome for an affected population to the change in outcomes for an unaffected population. The design controls for baseline differences between the two groups as well as changing demographics to estimate the independent effect of the policy change. The difference-in-differences design also controls for secular trends that equally affect both the treated and untreated groups.

To ensure that there were no differences in trends in the outcome variables studied prior to the Medicaid expansion, an important underlying assumption in the validity of our differences-in-differences model, we analyzed the trends in Florida relative to California for the years 2011–2013 leading up to the expansion. We tested the parallel trends hypothesis statistically. In an event study framework, we allowed for different effects for each year in the pre period in both Florida and California by interacting the year dummies for 2011, 2012, and 2013 with an indicator for California. We did not find a statistically significant difference between the estimated time effects for Florida and California for any of the years in question, suggesting that while there was a difference in the outcome variable at baseline, the state-specific effects did not converge or diverge over time *before* the implementation of the Medicaid expansion.

In a sensitivity analysis, we examined STEMI patients separately because practice guidelines are clearer for STEMI, as it is a more severe form of AMI and patients with STEMI should be treated with expedient PCI. Therefore, we would expect that the policy change would be less impactful for these patients due to strict guidelines on the need for and timing of invasive reperfusion interventions. All analyses were conducted using Stata software, version 15 (College Station, TX). The University of California, San Francisco Institutional Review Board approved this study.

## Results

We identified 32,540 hospital admissions in California, which expanded Medicaid, and 23,451 in Florida, which did not, for a total of 55,991 hospital admissions for AMI that met inclusion criteria (Table 1). There were no significant changes in the number of patients included by calendar year (S1 Fig). More patients in Florida were non-Hispanic white than in California (42% CA vs 58% FL). Both states had a similar proportion of female patients (29% CA and 30% FL), and a similar mean age. A higher proportion of patients in California had STEMI (41% CA vs 36% FL) with a higher likelihood of documented comorbidities of heart failure (26% CA vs 22% FL) and renal failure (11% CA vs 8% FL).

**Table 1:** Summary statistics of patient characteristics by state.

	California			Florida		
	Overall	Pre-expansion	*Post-expansion	Overall	Pre-2014	*Post-2014
**Total observations**	32,540	11,784	20,756	23,451	16,623	6,828
**Age: mean (SD)**	52.6 (8.9)	52.2 (9)	52.8 (9)	51.9 (8.8)	51.9 (8.8)	52 (9)
**Race (%)**						
**White**	42	44	41	58	59	60
**Black**	12	12	12	18	17	18
**Hispanic**	30	30	30	19	19	18
**Other**	16	14	17	5	5	4
**STEMI (%)**	41	44	40	36	37	34
**Selected comorbidities at presentation (%)**						
Heart failure	26	24	26	22	22	22
Cardiac arrhythmias	22	21	22	23	23	23
Renal failure	11	10	11	8	7	9
Diabetes	31	31	31	27	28	27
**Initially presented to a non-PCI hospital (%)**	15	18	14	8	8	7
**PCI performed (%)**	63	63	63	67	67	66
**PCI performed within 48 hours (%)**	35	33	36	44	44	44
**30-day readmission (%)**	12	13	12	11	12	11
**In-hospital mortality (%)**	3	4	3	3	3	3

Abbreviations: SD–standard deviation; STEMI–ST-elevation myocardial infarction; PCI–Percutaneous coronary intervention

*In California, all counties expanded Medicaid by 2014. However, counties in the state began expanding Medicaid eligibility as early as 2011 and discharges were categorized as “post-reform” if they occurred during or after the month that county expanded Medicaid.

**Fig 1:**
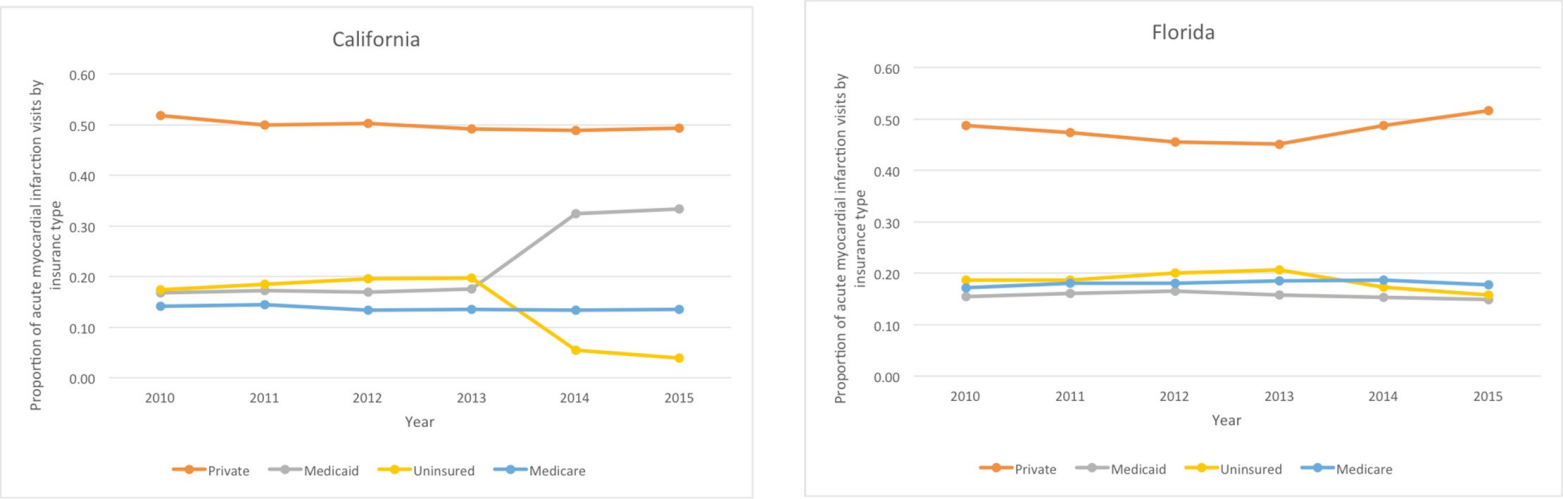
Proportion of acute myocardial infarction hospitalizations by insurance type, Medicaid or uninsured. The orange lines show trends in proportion of patients with private insurance, the grey lines show trends in patients insured by Medicaid, the yellow lines show trends in proportion of patients who are uninsured, and the blue lines show trends in proportion of patients who are insured by Medicare among patients hospitalized with AMI in California, on the left, and Florida, on the right.

Fig 1 shows the proportion of Medicaid, uninsured, Medicare, and privately insured patients admitted with AMI during the study period. Medicaid and uninsured patients were included in the study. The proportion of AMI patients in this sample with Medicaid insurance increased in California from 49% in 2010 to 90% in 2015, and the proportion of uninsured patients decreased from 51% to 10% over the years studied. In contrast, the proportion of AMI patients in Florida with Medicaid increased from 45% to 49% and the proportion of uninsured patients decreased just 4 percentage points from 55% to 51% in 2015.

**Fig 2:**
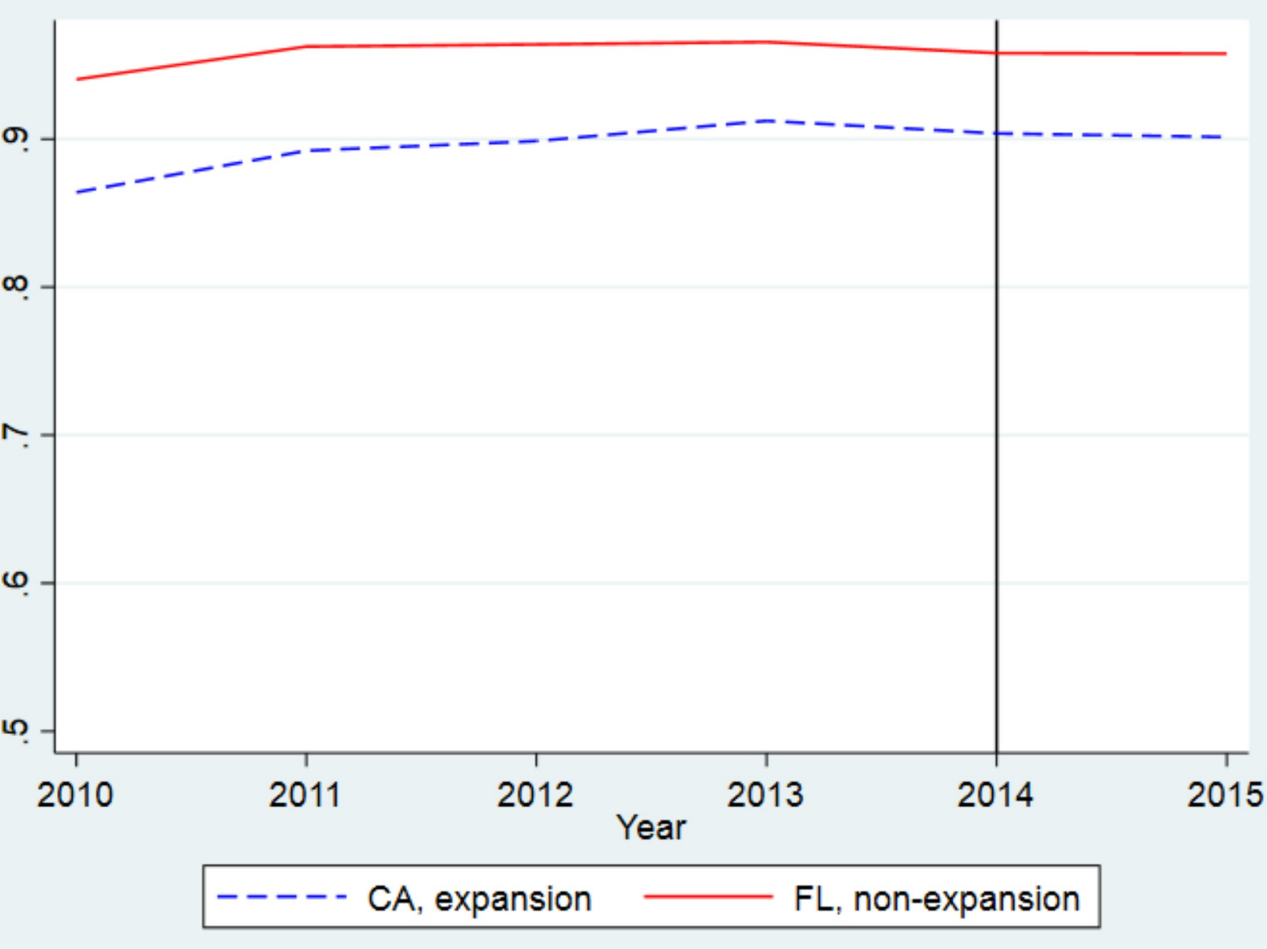
Unadjusted trends in likelihood of admission to a PCI hospital in California and Florida. The blue line shows trends in California, the red line shows trends in Florida. The y-axis shows the proportion of patients with AMI who were ultimately admitted to a PCI hospital. While all California counties expanded Medicaid by January 2014, as indicated by the black vertical line, some California counties began expanding Medicaid at earlier dates, beginning as early as 2011.

**Fig 3:**
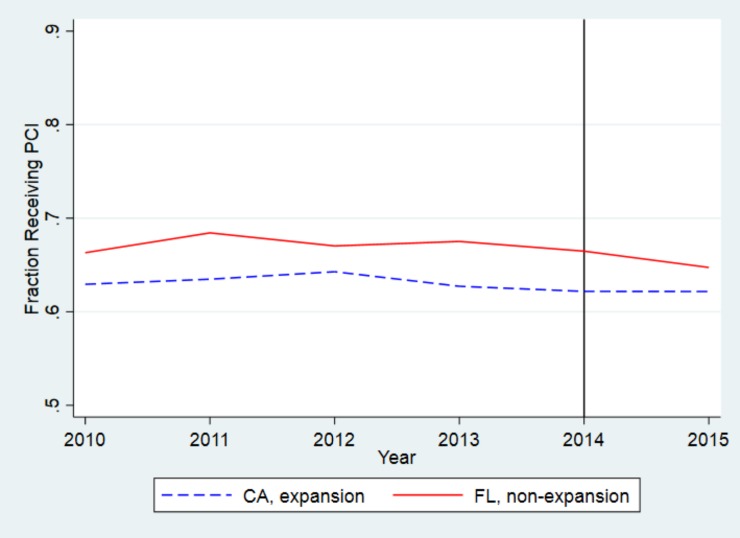
Unadjusted trends in likelihood of receiving PCI, California and Florida. The blue line shows trends in California, the red line shows trends in Florida. The y-axis shows the proportion of patients admitted with AMI who received PCI during index hospitalization. While all California counties expanded Medicaid by January 2014, as indicated by the black vertical line, some California counties began expanding Medicaid at earlier dates, beginning as early as 2011.

Fig 2 reveals the unadjusted trends in overall rates of admission to PCI hospitals, including patients who were transferred, in California and Florida. While patients in both states had high rates of access to PCI hospitals, Florida had higher rates of admission to PCI hospitals for all years studied, and rates remained fairly stable in both states throughout the study period. Fig 3 shows unadjusted trends in overall rates of PCI in California and Florida. Rates of PCI were higher in Florida than in California for all years studied. Trends in all outcome variables were similar prior to the beginning of the Medicaid expansion for both California and Florida.

**Fig 4:**
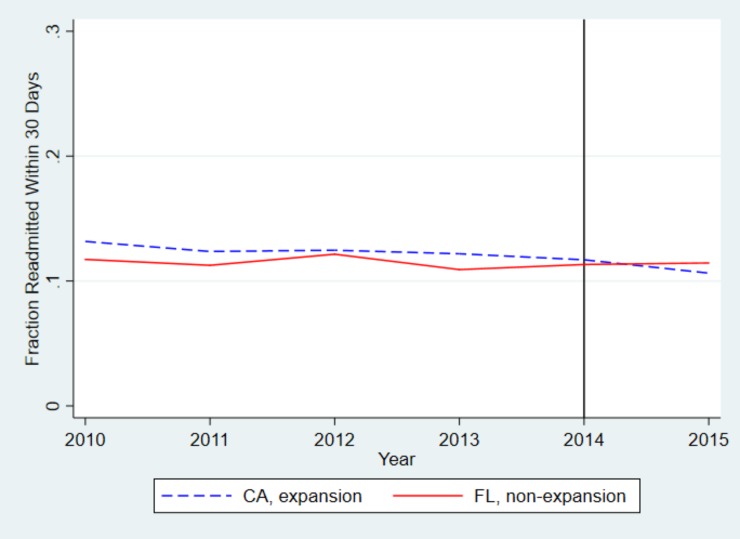
Unadjusted trends in likelihood of 30-day hospital readmission, California and Florida. The blue line shows trends in California, the red line shows trends in Florida. While all California counties expanded Medicaid by January 2014, as indicated by the vertical black line, some California counties began expanding Medicaid at earlier dates, beginning as early as 2011.

Fig 4 illustrates the proportion of patients readmitted to the hospital within 30 days of discharge from 2010 to 2015. We found that overall, the percentage of patients readmitted within 30 days in California has been higher than in Florida; however, after 2014, the percentage of patients readmitted within 30 days decreased in California to be lower than that in Florida.

In our regression analysis (Table 2), we found that 30-day readmission rates decreased by an absolute difference of 1.22 percentage points after the Medicaid expansion (95% CI -2.14 to -0.30, P < 0.01) after all AMI and by 1.56 percentage points (95% CI -2.98 to -0.14, P < 0.05) after STEMI in expansion areas versus non-expansion areas. Baseline 30-day readmission rates in California were 12.8% for AMI patients and 10.9% for STEMI patients, corresponding to a relative decrease in readmission rates of 9.5% after AMI and 14.3% after STEMI after Medicaid expansion. We did not find any significant changes in the likelihood of admission to a PCI hospital, transfer to a PCI hospital, receiving PCI, receiving early PCI, or in-hospital mortality after the Medicaid expansion in both samples of all AMI patients and STEMI patients only. Full regression results are available in S2 Table.

**Table 2:** The association of medicaid expansion with AMI outcomes: difference-in-difference regression results.

Outcome	Difference-in-Difference	95% confidence interval
**Likelihood of being transferred, if initially presented to a non-PCI hospital**	1.15	-5.3, 7.6
**Likelihood of ultimately being treated at a PCI hospital**	0.84	-1.56, 3.24
**Likelihood of PCI after AMI**	1.36	-.39, 3.1
**Likelihood of PCI within 48 hours of admission after AMI**	-0.17	-2.34, 2.0
**30-day hospital readmission, after admission with AMI**	-1.22**	-2.14, -0.30
**In-hospital mortality after AMI**	-0.38	-0.89, 0.14
**Likelihood of being transferred, if initially presented to a non-PCI hospital, patients with STEMI**	-9.26	-20.66, 2.14
**Likelihood of ultimately being treated at a PCI hospital, patients with STEMI**	1.36	-0.93, 3.65
**Likelihood of PCI, patients with STEMI**	1.48	-0.29, 3.24
**Likelihood of PCI within 48 hours of admission, STEMI patients**	0.05	-2.94, 3.05
**30-day hospital readmission, STEMI patients**	-1.56*	-2.98, -0.14
**In-hospital mortality, STEMI patients**	-0.46	-1.32, 0.39

* p<0.05

** p<0.01

Difference-in-difference represents the change from before to after expansion, as measured in absolute percentage points, in Medicaid expansion areas versus non-expansion areas

Abbreviations: PCI–percutaneous coronary intervention; AMI–acute myocardial infarction; STEMI–ST-elevation myocardial infarction

## Discussion

Overall, we found that 30-day hospital readmissions decreased by 9.5% after AMI, but that Medicaid expansion in California was not necessarily associated with improved access to PCI hospitals (whether by direct admission or transfer), treatment as defined by receipt of PCI (either during the hospital admission nor within 48 hours), or in-hospital mortality. The decrease in hospital readmissions persisted, and in fact, was greater, when restricted to a subset of patients with STEMI, who are typically sicker than the overall AMI population.

Our findings are consistent with a prior study of AMI care in expansion and non-expansion states that demonstrated a decrease in rates of uninsured patients with AMI but no change in rates of PCI or in-hospital mortality.[20] We built on these findings by also examining access to PCI hospitals and readmission rates. Though we focused on two states, our study used state-level databases to capture all of the patients in the states studied,[20] improving the generalizability of our findings. We also followed patients who were transferred, an important addition, as inter-hospital transfer remains an important aspect of access to care for a significant proportion of patients with AMI.

If insurance has previously been shown to be associated with improved access, treatment, and outcomes, why did our study show only improvement in 30-day readmissions but nothing else? There are several possibilities. First, the young, urban patient population we studied had high rates of access (85% on initial presentation in California, and 92% on initial presentation in Florida) to PCI hospitals, which is higher than the national average among older, Medicare patients.[44] This may partially explain why we did not identify an association between the Medicaid expansion and access to PCI hospitals or rates of PCI; perhaps the immediate management of AMI for this population was less likely to be influenced by insurance coverage than has been previously demonstrated. Additionally, because both being uninsured and having Medicaid insurance (as opposed to private insurance) are associated with decreased access to care and rates of invasive treatments for AMI,[25, 45] our findings may suggest that the expansion of Medicaid alone may not affect immediate access to care and treatments in the uninsured and Medicaid population. In other words, not all types of insurance may be seen equally; as other studies have found, patients with Medicaid receive different treatment from those with commercial private insurance.[25, 46]

Ours is the first study that we know of to examine whether the Medicaid expansion affected readmission rates after hospitalization for AMI. The reduction in 30-day readmission rates after AMI that we identified adds to a growing body of literature that has shown some improvements in care from the inpatient to outpatient settings after the Medicaid expansion.[15, 17, 18, 43] It is possible that the Medicaid expansion affected the outpatient environment,[15, 17, 18, 43, 47–49] such as through increased access to follow-up care for AMI patients, which prevented the need for readmission. These findings suggest that for Medicaid and uninsured patients, who tend to have higher readmission rates after AMI relative to privately insured patients, expanding Medicaid eligibility may mitigate these risks.[25, 50] Another important component of the ACA, the Hospital Readmissions Reduction Program, penalized hospitals with higher-than-expected readmission rates for specific conditions, which included AMI. While ongoing during the study period, we do not believe this affected our results as the policy was applied nationwide to both expansion and non-expansion states. The program was observed to be associated with a decrease in readmission rates for these conditions to 17.8% from 21.5%.[51] In this context, our finding of a 1.22 percentage point absolute reduction in readmissions for AMI after Medicaid expansion, which represents a relative reduction of 9.5%, is considered clinically significant.

As debate continues over whether to expand Medicaid in the 14 states where Medicaid has not yet been expanded, our study adds important additional information about the ways that Medicaid expansion affects care and outcomes after AMI. When it comes to acute treatments and outcomes after AMI for vulnerable patient populations, Medicaid expansion alone might not be enough to change outcomes and policymakers in all states should be open to additional potential interventions. However, policymakers in states that have not yet expanded Medicaid should consider the ways that expanding Medicaid might lead to improved outcomes after hospital discharge for the large population of low-income patients with AMI.

### Limitations

Our study included several limitations. First, our findings may be due to influences on practice patterns separate from the Medicaid expansion or on changing composition of our sample. Though our model carefully controls for comorbidities and changing demographics, there may be trends in our population not accounted for by our model. However, the use of eight different California county cohorts grouped based on the different times of expansion makes this unlikely, as these influences would have to impact practice patterns separately in different California counties as the Medicaid expansion was enacted at different times in different counties, which our model takes into account. Second, while California and Florida are two large, heavily populated, and ethnically and geographically diverse states, they are not necessarily representative of the entire US population. Of the states that did not expand Medicaid, Florida is one of just several states that included patient-level variables to allow for tracking patients after they are transferred or when they are readmitted in the HCUP datasets, which was essential for our analyses, and it was selected for its large population of uninsured individuals and demographic diversity. While this does not affect its internal validity because of our robust study design using difference-in-differences, this limits the generalizability of the study. Both California and Florida have a smaller proportion of the population that is non-Hispanic white (36.8%, California, and 53.5%, Florida) compared with the United States overall (60.4%). A higher proportion of the population in both California and Florida are Hispanic (29.3%, California, and 26.1%, Florida) compared with the United States overall (18.3%). Finally, Florida’s population is older (20.5% 65 years or older) than the United States overall (16%) and California (14.3%).[52] Third, we selected PCI as the treatment of interest because its use has previously been associated with insurance coverage,[25, 35, 36] and because it is an expensive, invasive, and often emergent procedure potentially influenced by a change in insurance coverage. However other aspects of care after AMI are also important, including optimal medical therapy and outpatient follow-up. Indeed, our finding that readmission rates decreased after the Medicaid expansion may be related to changes in access to care outside of the hospital; however, we were not able to track these types of health utilization with our data. Finally, although in-hospital mortality is an important outcome, we were unable to track out-of-hospital deaths with our data, since vital statistics linked to our patient data set were not yet available. This is important since previous literature shows that out-of-hospital mortality is often more affected than in-hospital death due to access to timely care,[53–55] thereby limiting the conclusions that can be drawn about the influence of the Medicaid expansion on mortality after AMI in our study and in others that have not found an association between the Medicaid expansion and in-hospital mortality rates after AMI. [20],[40]

## Conclusions

We observed that Medicaid expansion was associated with a decrease in 30-day readmissions after AMI, but not other outcomes of access to PCI hospitals, treatments received, or in-hospital mortality. Our findings suggest that the expansion of Medicaid alone may not affect access to emergent care and treatments immediately after AMI but may affect outcomes after hospital discharge in this vulnerable patient population.

## Supporting information

S1 Fig (PDF)

S1 Table (DOCX)

S2 Table (DOCX)
