# Supplementary material for: Effects of Medicaid expansion on access, treatment and outcomes for patients with acute myocardial infarction
Source: PLoS One. 2020 Apr 23;15(4):e0232097. doi: 10.1371/journal.pone.0232097 (PMC7179915; doi:10.1371/journal.pone.0232097)
Supplement: S1 Fig — (PDF) [file pone.0232097.s001.pdf]

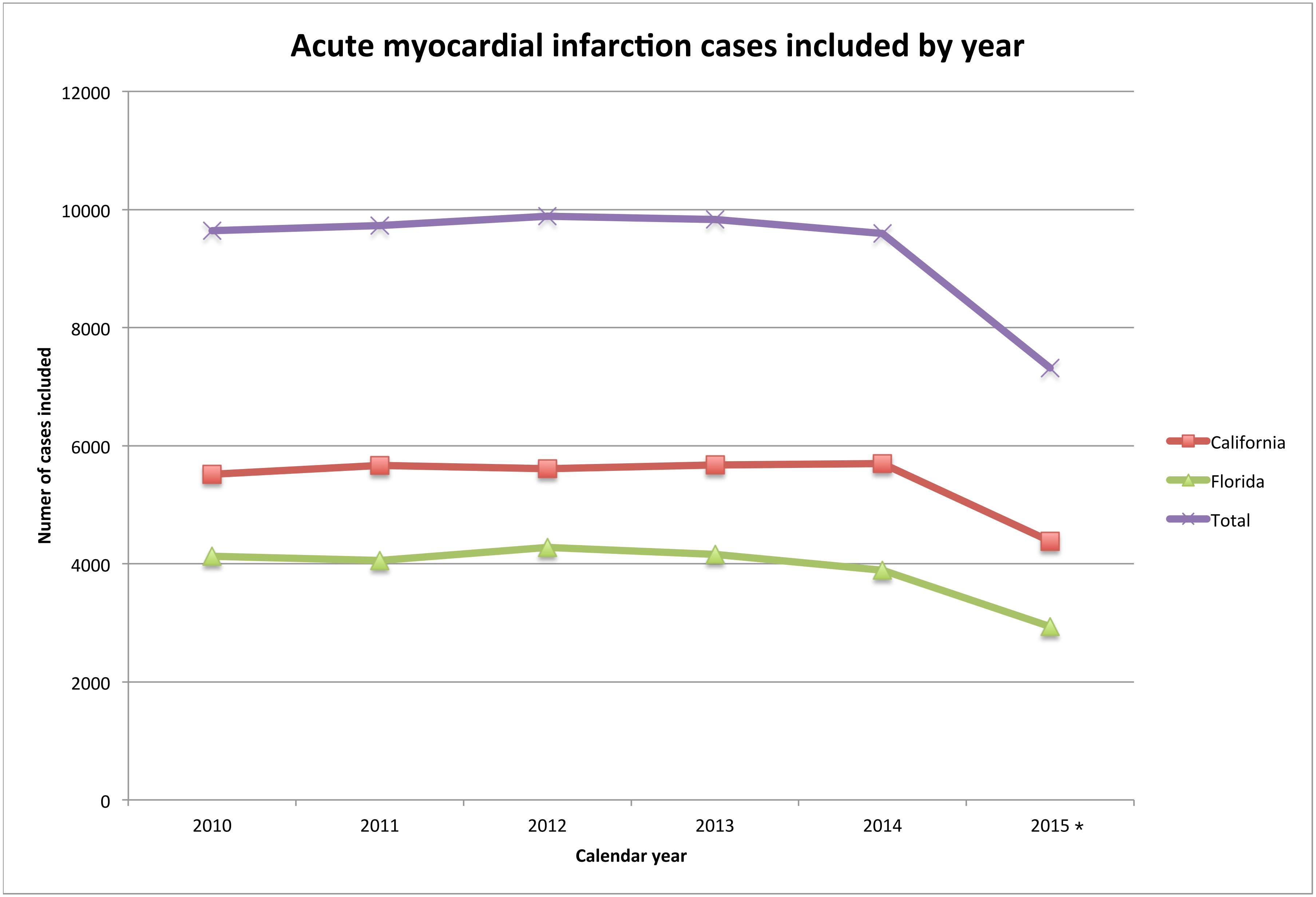

*AMI cases from October, November, and December 2015 were not included due to the transition to ICD-10-CM on October 1, 2015
