# Supplementary material for: Effects of Medicaid expansion on access, treatment and outcomes for patients with acute myocardial infarction
Source: PLoS One. 2020 Apr 23;15(4):e0232097. doi: 10.1371/journal.pone.0232097 (PMC7179915; doi:10.1371/journal.pone.0232097)
Supplement: S1 Table — (DOCX) [file pone.0232097.s002.docx]

| County | Date of expansion |
| --- | --- |
| Alameda | Jul-11 |
| Alpine | Jan-12 |
| Amador | Jan-12 |
| Butte | Jan-12 |
| Calaveras | Jan-12 |
| Colusa | Jan-12 |
| Contra Costa | Jul-11 |
| Del Norte | Jan-12 |
| El Dorado | Jan-12 |
| Fresno | January 2014 (No early expansion) |
| Glenn | Jan-12 |
| Humboldt | Jan-12 |
| Imperial | Jan-12 |
| Inyo | Jan-12 |
| Kern | Jul-11 |
| Kings | Jan-12 |
| Lake | Jan-12 |
| Lassen | Jan-12 |
| Los Angeles | Jul-11 |
| Madera | Jan-12 |
| Marin | Jan-12 |
| Mariposa | Jan-12 |
| Mendocino | Jan-12 |
| Merced | January 2014 (No early expansion) |
| Modoc | Jan-12 |
| Mono | Jan-12 |
| Monterey | Mar-13 |
| Napa | Jan-12 |
| Nevada | Jan-12 |
| Orange | Jul-11 |
| Placer | Aug-12 |
| Plumas | Jan-12 |
| Riverside | Jan-12 |
| Sacramento | Nov-12 |
| San Benito | Jan-12 |
| San Bernadino | Jan-12 |
| San Diego | Jul-11 |
| San Francisco | Jul-11 |
| San Joaquin | Jun-12 |
| San Luis Obispo | January 2014 (No early expansion) |
| San Mateo | Jul-11 |
| Santa Barbara | January 2014 (No early expansion) |
| Santa Clara | Jul-11 |
| Santa Cruz | Jan-12 |
| Shasta | Jan-12 |
| Sierra | Jan-12 |
| Siskiyou | Jan-12 |
| Solano | Jan-12 |
| Sonoma | Jan-12 |
| Stanislaus | January 2014 (No early expansion) |
| Sutter | Jan-12 |
| Tehama | Jan-12 |
| Trinity | Jan-12 |
| Tulare | Mar-13 |
| Tuolumne | Jan-12 |
| Ventura | Jul-11 |
| Yolo | Jul-12 |
| Yuba | Jan-12 |
