# Supplementary material for: Effects of Medicaid expansion on access, treatment and outcomes for patients with acute myocardial infarction
Source: PLoS One. 2020 Apr 23;15(4):e0232097. doi: 10.1371/journal.pone.0232097 (PMC7179915; doi:10.1371/journal.pone.0232097)
Supplement: S2 Table — (DOCX) [file pone.0232097.s003.docx]

|  | **Likelihood of admission to PCI hospital [95% confidence interval]** | **Likelihood of transfer, initial hospital not a PCI hospital [95% confidence interval]** | **Likelihood of PCI [95% confidence interval]** | **Likelihood of PCI within 48 hours [95% confidence interval]** | **Readmission within 30 days [95% confidence interval]** | **In-hospital mortality [95% confidence interval]** |
| --- | --- | --- | --- | --- | --- | --- |
| Difference-in-differences: effect of Medicaid expansion | 0.01 [-0.02,0.03] | 0.01[-0.05,0.08] | 0.01 [-0.00,0.03] | 0 [-0.02,0.02] | -0.01** [-0.02,-0.00] | 0 [-0.01,0.00] |
| Age in years at admission | 0.02*[0.00,0.03] | 0.06**[0.02,0.09] | 0 [-0.01,0.02] | 0.10** [0.09,0.12] | 0.01* [0.00,0.02] | 0.00+ [-0.00,0.01] |
| age^2 | -0.00* [-0.00,-0.00] | -0.00* [-0.00,-0.00] | 0 [-0.00,0.00] | -0.00** [-0.00,-0.00] | -0.00+ [-0.00,0.00] | 0 [-0.00,0.00] |
| age^3 | 0.00+ [-0.00,0.00] | 0[-0.00,0.00] | 0 [-0.00,0.00] | 0.00** [0.00,0.00] | 0 [-0.00,0.00] | 0 [-0.00,0.00] |
| Female sex | -0.02**[-0.03,-0.01] | -0.03*[-0.06,-0.00] | -0.05** [-0.06,-0.04] | -0.07** [-0.08,-0.06] | 0.02** [0.01,0.02] | 0.01** [0.00,0.01] |
| Rural | 0.05 [-0.04,0.15] | 0.07[-0.20,0.33] | 0.16** [0.09,0.23] | -0.18** [-0.28,-0.08] | -0.05** [-0.08,-0.03] | 0 [-0.01,0.01] |
| Race: black | -0.02* [-0.04,-0.00] | -0.12** [-0.18,-0.06] | -0.08** [-0.10,-0.06] | -0.07** [-0.09,-0.05] | 0.02** [0.01,0.03] | 0 [-0.01,0.00] |
| Race: Hispanic | -0.02* [-0.04,-0.00] | -0.05 [-0.12,0.02] | -0.05** [-0.06,-0.03] | -0.06** [-0.08,-0.04] | 0 [-0.01,0.01] | 0.00* [0.00,0.01] |
| Race: other | 0.01 [-0.00,0.02] | 0.04 [-0.02,0.10] | -0.02* [-0.03,-0.00] | -0.01 [-0.03,0.01] | -0.01** [-0.02,-0.00] | 0.01* [0.00,0.01] |
| STEMI | 0.06** [0.05,0.08] | -0.03 [-0.08,0.02] | 0.38** [0.36,0.40] | 0.28** [0.25,0.30] | -0.01** [-0.02,-0.01] | 0.01** [0.01,0.02] |
| Congestive Heart Failure | -0.01* [-0.02,-0.00] | -0.03+ [-0.06,0.00] | -0.12** [-0.13,-0.11] | -0.10** [-0.11,-0.09] | 0.06** | 0.01** [0.01,0.01] |
| Cardiac Arrhythmias | 0.01** [0.00,0.02] | -0.02 [-0.06,0.01] | -0.04** [-0.05,-0.03] | 0.02** [0.01,0.03] | 0.01** [0.01,0.02] | 0.03** [0.03,0.03] |
| Valvular Disease | 0.01 [-0.00,0.02] | 0.09** [0.04,0.14] | -0.08** [-0.09,-0.06] | -0.07** [-0.08,-0.05] | 0.02** [0.01,0.03] | -0.01+ [-0.01,0.00] |
| Pulmonary Circulation Disorders | 0 [-0.02,0.02] | 0.04 [-0.03,0.11] | -0.06** [-0.09,-0.04] | -0.05** [-0.07,-0.03] | 0.01 [-0.01,0.03] | 0.02** [0.01,0.03] |
| Peripheral Vascular Disorders | 0.03** [0.02,0.04] | 0.11** [0.05,0.16] | -0.02** [-0.04,-0.01] | -0.04** [-0.05,-0.03] | 0.04** [0.02,0.05] | 0.01** [0.01,0.02] |
| Hypertension, Uncomplicated | 0.01** [0.00,0.01] | 0.03* [0.00,0.06] | 0 [-0.01,0.01] | 0.01** [0.00,0.02] | 0.02** [0.01,0.02] | -0.02** [-0.03,-0.02] |
| Paralysis | -0.02 [-0.06,0.02] | -0.01 [-0.11,0.09] | -0.01 [-0.05,0.03] | -0.17** [-0.21,-0.14] | 0.01 [-0.02,0.04] | 0.02 [-0.01,0.06] |
| Other Neurological Disorders | -0.04** [-0.06,-0.02] | -0.13** [-0.18,-0.08] | -0.04** [-0.06,-0.03] | -0.06** [-0.08,-0.04] | 0 [-0.01,0.01] | 0.16** [0.15,0.18] |
| Chronic Pulmonary Disease | -0.01** [-0.02,-0.00] | -0.03+ [-0.07,0.00] | -0.05** [-0.06,-0.04] | -0.06** [-0.08,-0.05] | 0.03** [0.02,0.04] | -0.01* [-0.01,-0.00] |
| Diabetes, Uncomplicated | 0 [-0.01,0.01] | 0.03+ [-0.00,0.05] | 0 [-0.01,0.01] | 0 [-0.01,0.01] | 0.03** [0.02,0.03] | 0.00+ [-0.00,0.01] |
| Diabetes, Complicated | 0 [-0.02,0.01] | 0 [-0.05,0.05] | -0.04** [-0.06,-0.02] | -0.05** [-0.06,-0.03] | 0.06** [0.04,0.07] | -0.01** [-0.02,-0.00] |
| Hypothyroidism | 0 [-0.01,0.01] | 0 [-0.05,0.05] | 0 [-0.02,0.02] | -0.01 [-0.03,0.01] | 0 [-0.01,0.02] | -0.01** [-0.02,-0.00] |
| Renal Failure | -0.03* [-0.05,-0.00] | -0.06+ [-0.13,0.00] | -0.02+ [-0.05,0.00] | -0.05** [-0.07,-0.03] | 0.06** [0.04,0.08] | 0.01* [0.00,0.02] |
| Liver Disease | -0.02** [-0.04,-0.01] | -0.05* [-0.10,-0.00] | -0.03** [-0.05,-0.01] | -0.03** [-0.05,-0.01] | 0.03** [0.01,0.05] | 0.08** [0.07,0.10] |
| Peptic Ulcer Disease Excluding Bleeding | -0.04+ [-0.07,0.00] | -0.09 [-0.21,0.02] | -0.04 [-0.09,0.01] | -0.08** [-0.12,-0.03] | 0.04* [0.00,0.08] | -0.01 [-0.03,0.01] |
| AIDS/HIV | 0.03+ [-0.00,0.06] | 0.04 [-0.15,0.24] | 0.01 [-0.05,0.06] | -0.02 [-0.08,0.04] | 0.04 [-0.01,0.08] | 0 [-0.03,0.02] |
| Lymphoma | -0.03 [-0.09,0.02] | -0.05 [-0.24,0.13] | 0.05 [-0.04,0.13] | -0.03 [-0.12,0.05] | 0.05 [-0.01,0.12] | 0 [-0.03,0.04] |
| Metastatic Cancer | -0.04+ [-0.08,0.01] | -0.11 [-0.24,0.02] | -0.10** [-0.15,-0.04] | -0.12** [-0.18,-0.07] | 0.04 [-0.02,0.10] | 0.07** [0.03,0.12] |
| Solid Tumor Without Metastasis | -0.01 [-0.04,0.02] | -0.15** [-0.25,-0.05] | -0.05** [-0.09,-0.01] | -0.10** [-0.14,-0.06] | 0.06** [0.02,0.10] | 0.02 [-0.00,0.04] |
| Rheumatoid Arthritis/Collagen Vascular | 0.01 [-0.01,0.04] | 0.02 [-0.07,0.11] | -0.03+ [-0.06,0.01] | 0 [-0.03,0.03] | 0.04** [0.01,0.08] | -0.01 [-0.02,0.00] |
| Coagulopathy | 0.02** [0.01,0.04] | 0.11** [0.05,0.17] | -0.11** [-0.13,-0.08] | -0.11** [-0.13,-0.09] | 0.01 [-0.01,0.02] | 0.03** [0.02,0.04] |
| Obesity | 0.01 [-0.00,0.02] | 0.02 [-0.02,0.06] | 0 [-0.01,0.01] | 0 [-0.01,0.02] | -0.01* [-0.02,-0.00] | -0.00* [-0.01,-0.00] |
| Weight Loss | -0.06** [-0.10,-0.02] | -0.09** [-0.16,-0.02] | -0.06** [-0.09,-0.03] | -0.09** [-0.12,-0.06] | 0.05** [0.03,0.07] | 0 [-0.01,0.02] |
| Fluid and Electrolyte Disorders | -0.01+ [-0.02,0.00] | -0.18** [-0.22,-0.13] | -0.07** [-0.08,-0.06] | -0.05** [-0.07,-0.04] | 0.01** [0.00,0.02] | 0.04** [0.03,0.05] |
| Blood Loss Anemia | -0.03 [-0.07,0.01] | -0.18** [-0.29,-0.06] | -0.11** [-0.16,-0.06] | -0.10** [-0.15,-0.06] | 0.03 [-0.01,0.07] | -0.03** [-0.05,-0.02] |
| Deficiency Anemia | -0.01 [-0.03,0.01] | -0.08* [-0.15,-0.00] | -0.05** [-0.09,-0.02] | -0.07** [-0.10,-0.04] | 0.01 [-0.02,0.03] | -0.02** [-0.03,-0.02] |
| Alcohol Abuse | -0.02** [-0.03,-0.01] | -0.09** [-0.13,-0.05] | -0.04** [-0.06,-0.03] | -0.06** [-0.08,-0.05] | 0 [-0.01,0.01] | -0.01** [-0.02,-0.01] |
| Drug Abuse | 0 [-0.01,0.01] | -0.10** [-0.13,-0.06] | -0.06** [-0.07,-0.04] | -0.01 [-0.02,0.01] | 0.02** [0.01,0.03] | -0.01** [-0.01,-0.00] |
| Psychoses | -0.13** [-0.20,-0.06] | -0.15** [-0.22,-0.08] | -0.03+ [-0.06,0.00] | -0.15** [-0.19,-0.12] | 0.06** [0.03,0.09] | -0.01* [-0.03,-0.00] |
| Depression | -0.03** [-0.05,-0.02] | -0.08** [-0.12,-0.03] | -0.05** [-0.06,-0.03] | -0.06** [-0.08,-0.05] | 0.03**[0.02,0.04] | -0.02**[-0.03,-0.02] |
| Hypertension, Complicated | 0 [-0.02,0.03] | 0.04 [-0.04,0.11] | -0.06** [-0.08,-0.03] | -0.04** [-0.06,-0.02] | 0.01+ [-0.00,0.03] | -0.03** [-0.04,-0.02] |
| Year 2011 | 0.02** [0.01,0.04] | -0.01 [-0.07,0.04] | 0.02* [0.00,0.03] | 0.03** [0.01,0.04] | 0 [-0.01,0.00] | 0 [-0.00,0.01] |
| Year 2012 | 0.03** [0.01,0.04] | 0.01 [-0.06,0.07] | 0.02* [0.00,0.03] | 0.05** [0.03,0.07] | 0 [-0.01,0.01] | 0 [-0.01,0.00] |
| Year 2013 | 0.04** [0.02,0.06] | -0.02 [-0.09,0.05] | 0.02** [0.01,0.04] | 0.05** [0.03,0.06] | 0 [-0.01,0.00] | 0 [-0.01,0.00] |
| Year 2014 | 0.03** [0.01,0.05] | -0.01 [-0.09,0.06] | 0.02* [0.00,0.04] | 0.05** [0.03,0.07] | -0.01 [-0.02,0.00] | 0 [-0.01,0.01] |
| Year 2015 | 0.03** [0.01,0.05] | 0 [-0.08,0.07] | 0.03** [0.01,0.05] | 0.05** [0.03,0.07] | -0.02**[-0.03,-0.00] | -0.02**[-0.03,-0.02] |
| County group 1* | -0.02 [-0.09,0.05] | -0.25* [-0.49,-0.02] | -0.03 [-0.08,0.02] | -0.09** [-0.16,-0.02] | 0 [-0.01,0.02] | 0 [-0.01,0.00] |
| County group 2* | -0.06** [-0.10,-0.02] | -0.15* [-0.31,-0.00] | -0.06** [-0.09,-0.03] | -0.05** [-0.09,-0.01] | 0.02** [0.01,0.03] | 0 [-0.01,0.00] |
| County group 3* | -0.11** [-0.19,-0.03] | -0.19* [-0.37,-0.01] | -0.08** [-0.12,-0.03] | -0.16** [-0.22,-0.11] | 0.02** [0.00,0.03] | 0 [-0.01,0.00] |
| County group 4* | -0.2 [-0.48,0.09] | -0.15 [-0.53,0.24] | -0.17* [-0.33,-0.01] | -0.25** [-0.33,-0.17] | -0.02+ [-0.04,0.00] | 0.01* [0.00,0.02] |
| County group 5* | -0.11 [-0.32,0.10] | -0.11 [-0.57,0.35] | 0 [-0.15,0.14] | -0.13+ [-0.28,0.02] | 0.03 [-0.03,0.09] | -0.01 [-0.04,0.02] |
| County group 6* | -0.04 [-0.15,0.06] | -0.23 [-0.65,0.19] | -0.07 [-0.17,0.02] | -0.11 [-0.28,0.07] | -0.02 [-0.06,0.01] | -0.02 [-0.04,0.01] |
| County group 7* | -0.03 [-0.12,0.05] | -0.05 [-0.47,0.37] | -0.04 [-0.13,0.05] | -0.06 [-0.15,0.03] | -0.01 [-0.02,0.01] | -0.01 [-0.01,0.00] |
| County group 8* | -0.11 [-0.30,0.09] | -0.18 [-0.58,0.22] | -0.07 [-0.16,0.02] | -0.14+ [-0.28,0.00] | -0.01* [-0.03,-0.00] | 0 [-0.01,0.02] |
| Constant | 0.58** [0.33,0.82] | -0.44+ [-0.95,0.08] | 0.56** [0.35,0.76] | -1.52** [-1.72,-1.31] | -0.11+ [-0.23,0.02] | -0.07* [-0.13,-0.00] |
| N | 55991 | 6834 | 55991 | 55991 | 55991 | 55991 |

*County group 1 -- No early expansion (county expanded Jan 2014); County group 2: County expanded July 2011; County group 3: County expanded Jan 2012; County group 4: County expanded June 2012; County group 5: County expanded July 2012; County group 6: County expanded Aug 2012; County group 7: County expanded Nov 2012; County group 8: County expanded Mar 2013
